# Long‐term outcomes in patients with normal coronary arteries, nonobstructive, or obstructive coronary artery disease on invasive coronary angiography

**DOI:** 10.1002/clc.23686

**Published:** 2021-07-03

**Authors:** Christopher A. Hanson, Edwin Lu, Saad S. Ghumman, Michelle L. Ouellette, Adrián I. Löffler, George A. Beller, Jamieson M. Bourque

**Affiliations:** ^1^ Department of Medicine, Cardiovascular Division University of Virginia Health System Charlottesville Virginia USA; ^2^ Department of Medicine University of North Carolina Health System Chapel Hill North Carolina USA; ^3^ Department of Radiology University of Virginia Health System Charlottesville Virginia USA

**Keywords:** cardiovascular outcomes, coronary angiography, coronary artery disease, nonobstructive coronary artery disease

## Abstract

**Background:**

Normal or near normal coronary arteries (NNCA) or nonobstructive coronary artery disease (CAD) are commonly found on invasive coronary angiography (ICA).

**Hypothesis:**

We aimed to determine long‐term outcomes by severity of CAD in a contemporary cohort of patients undergoing ICA for evaluation for ischemic heart disease.

**Methods:**

We assessed a consecutive cohort of 925 patients who underwent non‐emergent ICA over 24 months. Cardiac death (CD), nonfatal myocardial infarction (NFMI), late revascularization, and medication use were assessed.

**Results:**

Follow‐up data was available in 850 patients. Of patients without heart failure, at a median of 6.0 years, there was a significant decrease in survival free from CD or NFMI, and from all cardiac events, for those with obstructive CAD compared with patients with NNCAs or nonobstructive CAD (*p* < .001 for both). No differences between NNCA and nonobstructive CAD patients in rates of CD or NFMI (2.0% vs. 2.1%/year, *p* = .58) or all cardiac events (2.4% vs. 2.9%/year, *p* = .84) were observed.

**Conclusion:**

Long‐term follow‐up in a contemporary cohort of consecutive patients undergoing non‐emergent ICA for detection of CAD showed no difference in annual rates of CD or NFMI, or total cardiac events, in patients with NNCAs versus those with nonobstructive CAD, whereas patients with obstructive CAD had significantly more events. Event rates were low and similar by gender. Use of aspirin, lipid lowering therapy, and beta‐blockers increased in all subgroups after ICA. We speculate this may explain the low incidence of subsequent cardiac events, and similar event rates in patients with NNCA and nonobstructive CAD, even in patients presenting with non‐ST‐elevation MI.

## 1 INTRODUCTION

Normal or near normal coronary arteries (NNCA), or nonobstructive coronary artery disease (CAD), are found on average in 55% of patients referred for invasive coronary angiography (ICA) for suspected coronary artery disease.^1^ Similarly, in a recent study in which coronary computed tomography angiography (CCTA) was used for identification of patients with CAD, 58% had either NNCA or nonobstructive disease defined as 10%–50% stenosis.^2^ In that study, 21% had nonobstructive CAD defined as stenosis between 10% and 49% luminal narrowing. In the PROMISE trial, 20% of patients enrolled in the CCTA arm had nonobstructive CAD defined as 30%–50% stenosis.^3^ The death or nonfatal myocardial infarction (NFMI) rate has universally been reported to be higher in patients with nonobstructive CAD compared to patients found to have NNCA.^4^, ^5^, ^6^, ^7^, ^8^, ^9^, ^10^ The death or MI rate for patients with non‐ST‐elevation (NSTEMI) and nonobstructive CAD is reported to be 3.5 times higher than in NSTEMI patients with NNCA.^5^ Some prior reports have suggested that cardiac event rates are higher in women with nonobstructive CAD than in men.^11^, ^12^

We previously reported, in a cohort of 925 consecutive patients without known CAD referred for non‐emergent ICA after either a positive stress test, a NSTEMI, unstable or stable angina, and heart failure (HF), that 45% had either NNCA (0%–20% stenosis) or nonobstructive CAD (21%–49% stenosis).^13^ After 2 years of follow‐up in this clinically high‐risk group of patients, we found no difference in cardiac death (CD), NFMI or late revascularization between those with NNCA and those with nonobstructive CAD. As expected, event rates were significantly higher (6.7%/year) in patients with obstructive CAD (>50% stenosis). No differences in event rates by sex were observed.^13^ Because our follow‐up was limited in this initial report, we sought to determine long‐term outcomes in this patient population who were referred for an initial invasive evaluation for suspected CAD. We also specifically wanted to determine how changes in medical therapy after coronary anatomy was identified might have influenced long‐term outcomes. Finally, we sought to determine if there were any disparities in guideline‐recommended therapy between men and women in this cohort.

## 2 METHODS

**FIGURE 1:**
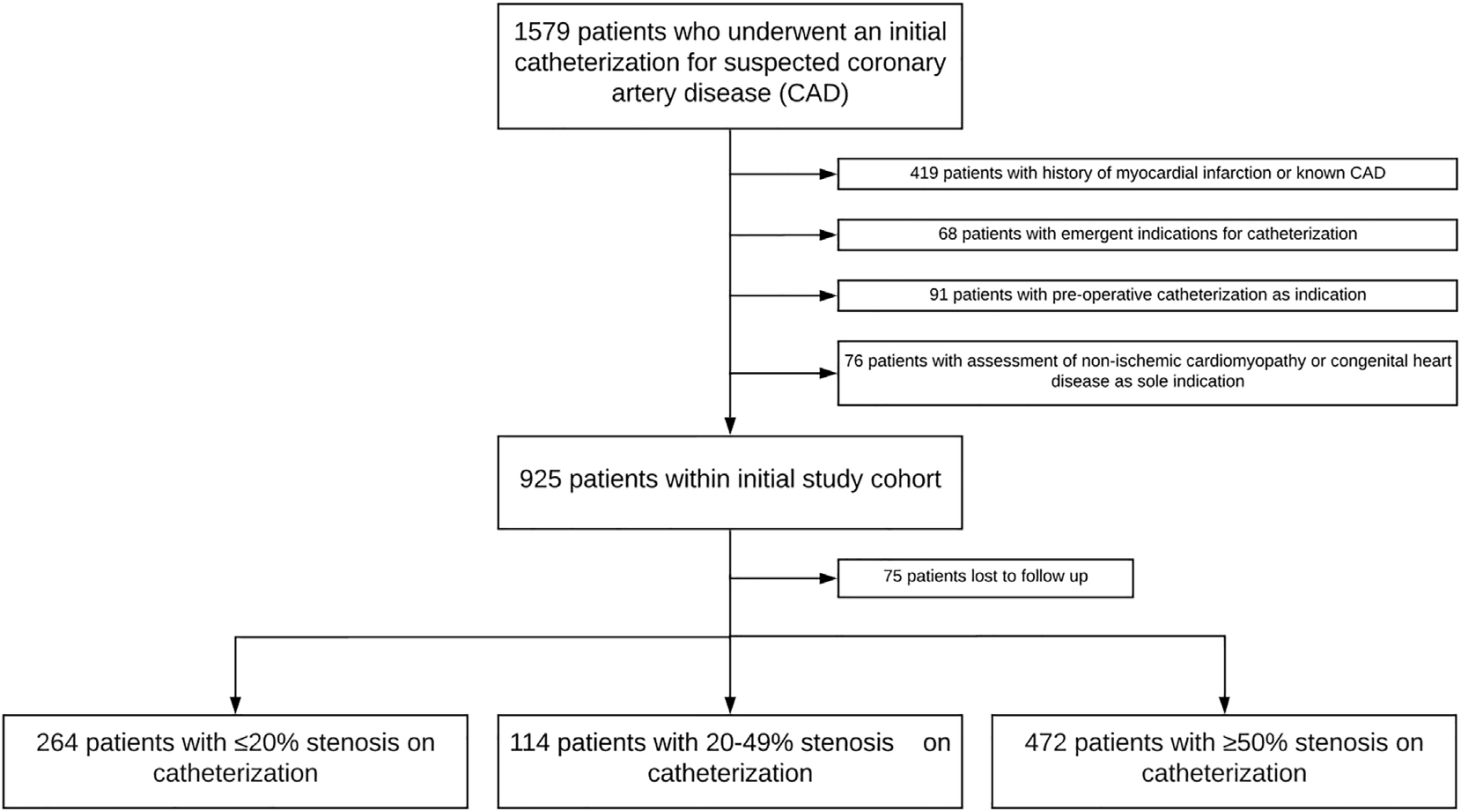
Patient flow diagram showing derivation of final study cohort at time of follow‐up including patient selection, excluded patients, patients lost to follow‐up, and the categorization of angiographic stenosis

As previously described in the initial short‐term follow‐up study,^13^ all patients who underwent an initial ICA at the University of Virginia for suspected CAD between January 1, 2012 and December 31, 2013 were reviewed (n = 1579). Patients with known CAD, history of myocardial infarction (MI), or emergent indications for ICA (such as ST‐elevation MI, cardiogenic shock, or post‐cardiac arrest) were excluded. ICAs performed for preoperative evaluation for transplant or cardiothoracic surgery or evaluation of non‐ischemic cardiomyopathy or congenital heart disease alone was also excluded. The final study cohort comprised of 925 patients. Figure 1 demonstrates the derivation of the final study cohort. The University of Virginia Institutional Review Board gave approval for the study protocol and rendered waiver of informed consent.

Clinical characteristics, such as patient demographics, medical comorbidities, medications, pertinent laboratory data, stress test findings, and cardiac imaging findings were entered into the medical record and reviewed. Ten‐year atherosclerotic cardiovascular disease (ASCVD) risk score was calculated based on the medical record. ASCVD risk score found to be ≥7.5% were considered elevated risk for cardiac events.^14^ Outcomes, including all‐cause mortality, CD, and cardiac events such as NFMI (NSTEMI or ST‐elevation MI) or coronary artery revascularization procedures were recorded. Outcomes were collected from the electronic medical record. If outcomes were uncertain or in subjects in whom follow‐up was unavailable from the medical record, telephone contact was made. CD was defined as any death with a cardiac cause, or without a clear noncardiac cause. A NSTEMI, with or without typical ischemic electrocardiographic changes in the setting of a history consistent with an acute coronary syndrome, was confirmed via elevated peak troponin I ≥ 10 times the upper limit of normal within 30 days preceding ICA. Late revascularization was designated as any coronary revascularization performed ≥4 weeks after the initial ICA (excluding planned interventions); revascularization procedures included percutaneous coronary interventions and coronary artery bypass grafting.

### 2.1 Stress testing, cardiac imaging, and ICA

Patients who were referred for ICA following an abnormal stress test had previously undergone exercise stress electrocardiography, exercise stress electrocardiography with myocardial perfusion imaging (MPI) with quantitative gated single‐photon emission computed tomography (SPECT), vasodilator stress MPI with SPECT or positron emission tomography (PET), exercise or dobutamine stress echocardiography, or vasodilator stress cardiac magnetic resonance imaging (cardiac MRI). All of the aforementioned exercise and pharmacological stress and imaging protocols were standardized consistent with guidelines. All of the studies were interpreted by experienced readers. Details regarding interpretation of these studies and the criteria for presence of inducible ischemia have been published previously.^13^, ^14^, ^15^, ^16^, ^17^, ^18^, ^19^ High‐risk ischemia observed on MPI was defined as ≥10% left ventricular (LV) ischemia.^20^

Likewise, ICA was performed using contemporary clinical protocols. The angiograms were reviewed by two experienced interventional cardiologists who were blinded to the initial reports written by the physician performing the catheterizations. Referral indications for ICA were undertaken through review of the available medical record. The degree of coronary artery stenosis into three categories: NNCAs, nonobstructive CAD, and obstructive CAD. NNCAs were defined as ≤20% stenosis in all coronary arteries.^1^, ^13^, ^21^, ^22^ The term “near normal” is used as it is difficult to distinguish normal coronary arteries from those with minimal atherosclerotic plaques via ICA alone.^13^ Nonobstructive CAD was defined by at least 1 coronary artery with a 21%–49%.^1^, ^21^, ^23^ Obstructive CAD was defined by at least 1 coronary artery with a ≥50% stenosis.^1^, ^21^, ^23^ In patients with stenosis between 21% and 69%, an experienced interventional cardiologist, who was blinded to all clinical data, retrospectively performed quantitative coronary analysis to minimize inter‐observer variability in evaluating intermediate stenosis and to group them as either nonobstructive CAD or obstructive CAD. If fractional flow reserve (FFR) was performed, stenoses with FFR measurements ≥0.8 were considered nonobstructive. If the FFR measurements were <0.80 the coronary anatomy was considered to be obstructive. Appropriateness of ICA in patients without NSTEMI from this cohort was reported previously in a research letter. For this cohort, ICA was classified as appropriate in 918 patients (99.2%).^24^ The seven patients with inappropriate indications for ICA all had NNCAs.

### 2.2 Statistical analysis

Continuous variables were presented as medians (25th, 75th percentiles) where appropriate. Categorical variables were given as percentages and compared using chi‐square analysis and Fisher's exact testing, where appropriate. Alpha level of significance was set at <0.05. Event rates were calculated through person‐years analysis. Values were adjusted for 1 person‐year of follow‐up to provide annualized event rates. The calculation of events per patient‐years was performed by the number of events divided by the amount of person‐time at risk for each event measured. Kaplan–Meier survival analysis was performed to assess rates of cardiac events across degrees of coronary stenosis, referral indications, and gender. We analyzed outcomes in the subgroup referred without HF separately from the entire cohort as patients with HF found to have normal or NNCA or nonobstructive CAD would be expected to have events driven by their cardiomyopathy rather than downstream ischemic disease. The relationship of sex to these outcomes was analyzed through Cox proportional hazards analysis. All statistical analyses were performed using SAS (version 9.3; SAS Institute Inc, Cary, NC) and MedCalc (version 14; MedCalc, Mariakerke, Belgium).

## 3 RESULTS

### 3.1 Baseline characteristics

Of the 925 patients who underwent an index ICA for assessment of CAD, long‐term follow‐up was available in 850 patients (91.9%). The baseline characteristics of the original 925 patients has been reported and analyzed previously.^13^ The baseline characteristics of the 850 patients with long‐term follow‐up are summarized in Table 1. The baseline characteristics of the 715 patients with long‐term follow‐up are summarized in Supplementary Table I. There were no statistically significant differences in any baseline characteristics between the initial 925 patients and the 850 patients comprising this follow‐up cohort (*p* > .05 for all clinical characteristics across all stenosis grades). Referral indications for ICA included: positive stress testing (32.6%), NSTEMI (26.8%), unstable angina (13.3%), HF (13.1%), stable angina (11.5%), and other (2.7%). On ICA, 264 (31.1%) of patients had NNCAs, 114 (13.4%) patients had nonobstructive CAD, and 472 (55.6%) of the patients had obstructive CAD.

**TABLE 1:** Study cohort baseline characteristics in total and subdivided by severity of angiographic coronary stenosis

Clinical characteristic	Total cohort, n (%)	Severity of coronary stenosis		
		≤20% (n [%])	21%–49% (n [%])	≥50% (n [%])
Total patients	850	264 (31.0)	114 (13.4)	472 (55.6)
Age, years^a^	63.7 (±11.5)	57.2 (±11.8)	64.4 (±10.0)	64.4 (±11.6)
Female	377 (44.4)	143 (54.2)	61 (53.5)	173 (36.7)
Caucasian	691 (81.3)	196 (74.2)	94 (82.5)	403 (85.4)
Diabetes mellitus	297 (34.9)	80 (30.3)	35 (30.7)	182 (38.6)
Hypertension	666 (78.4)	180 (68.2)	85 (74.6)	401 (85.0)
Hyperlipidemia	577 (67.9)	146 (55.3)	65 (57.0)	366 (77.5)
Tobacco use	279 (32.8)	83 (31.4)	31 (27.2)	165 (35.0)
BMI ≥ 30	396 (47.4)	141 (53.4)	50 (43.8)	205 (43.4)
Congestive heart failure	230 (27.1)	76 (28.8)	28 (24.6)	126 (26.7)
Peripheral vascular disease	102 (12.0)	14 (5.3)	8 (7.0)	80 (16.9)
Cerebrovascular disease	31 (3.6)	3 (1.1)	2 (1.8)	26 (5.5)
Chronic kidney disease grade ≥ 3	80 (9.5)	13 (4.9)	6 (5.3)	61 (12.9)
ASCVD risk ≥ 7.5%	549 (75.2)	134 (50.8)	73 (64.0)	342 (72.5)
Chest pain or anginal SOB	680 (80.0)	201 (76.1)	77 (67.5)	402 (85.2)

*Note:* Baseline characteristics of the study cohort. ASCVD risk was unable to be classified in 120 patients.

Abbreviations: ASCVD, atherosclerotic cardiovascular disease; BMI, body mass index.

^a^ Mean ± SD provided for continuous variables.

### 3.2 Outcomes

**FIGURE 2:**
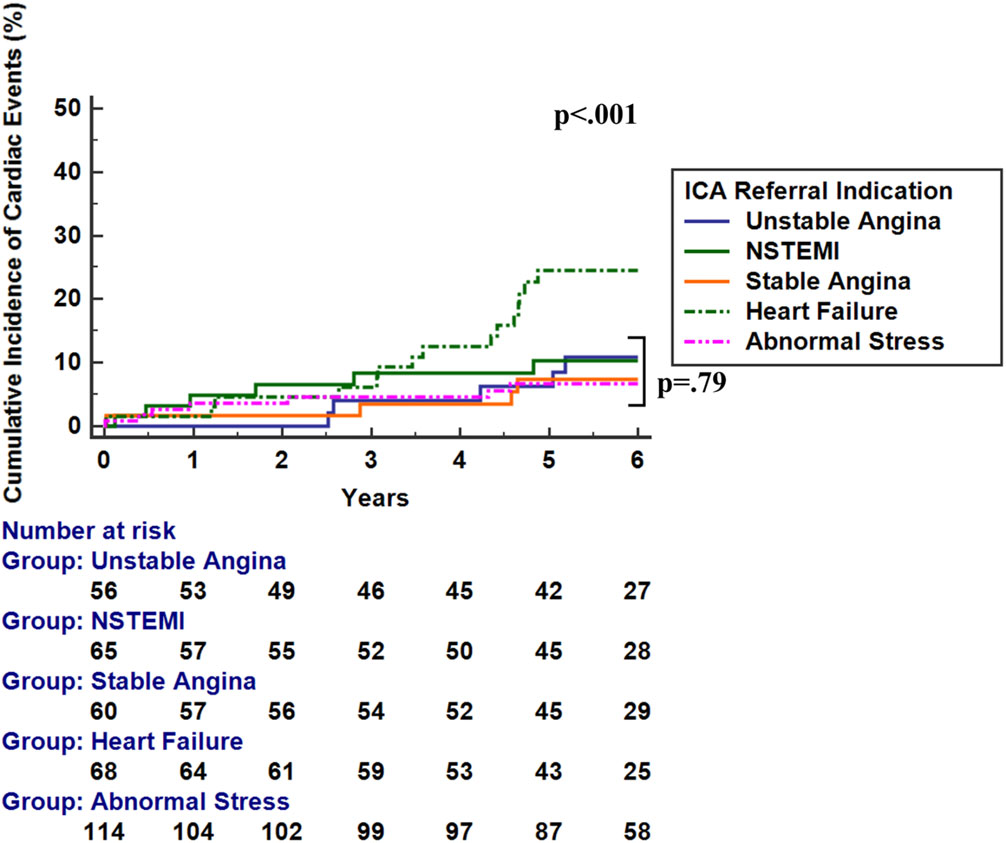
Cumulative events curves of freedom from cardiac events in patients with NNCA or nonobstructive CAD stratified by referral indication. Cardiac events include cardiac death, late revascularization, and NFMI. There was no observed difference in outcomes in patients with NNCA and nonobstructive CAD those referred for an abnormal stress test, NSTEMI, stable or unstable angina (*p* = .79 for all non‐HF referral indications). However, patients with HF with either NNCA or nonobstructive CAD had more cardiac events compared to the other referral subgroups (*p* < .001). CAD, coronary artery disease; HF, heart failure; NFMI, nonfatal myocardial infarction; NNCA, normal or near‐normal coronary arteries; NSTEMI, non‐ST‐elevation MI

Median follow‐up was 6.0 years. In those referred for abnormal stress, NSTEMI, or stable/unstable angina, Kaplan–Meier survival analysis showed a significant decrease in survival free from CD/NFMI, and total cardiac events for those with obstructive CAD compared to patients with NNCAs or nonobstructive CAD, *p* < .001. Surprisingly, there was still no difference in annual rates of CD/NFMI (1.6% vs. 1.9% per year, *p* = .44), or total cardiac events (1.7% vs. 2.2% per year, *p* = .61) observed between patients with NNCAs and nonobstructive CAD, respectively. Even in patients with NSTEMI, those with nonobstructive CAD did not experience an increase in cardiac events compared to patients with NNCA 2.6% vs. 2.0% (*p* = .82). Annual cardiac event rates were similar by gender over the follow‐up period by Kaplan–Meier survival analysis regardless of stenosis grade (*p* = .82 for NNCA; *p* = .50 for nonobstructive CAD; *p* = .20 for obstructive CAD). Event rates by gender and degree of coronary stenosis are summarized in Supplementary Table II. Kaplan–Meier survival analysis showed no difference in outcomes in the patients with NNCA versus nonobstructive CAD in those referred for an abnormal stress test, NSTEMI, HF, stable or unstable angina (*p* > .05 for all indications). Patients with HF with either NNCA or nonobstructive CAD had a worse prognosis than the other referral subgroups (*p* < .001) (Figure 2).

**FIGURE 3:**
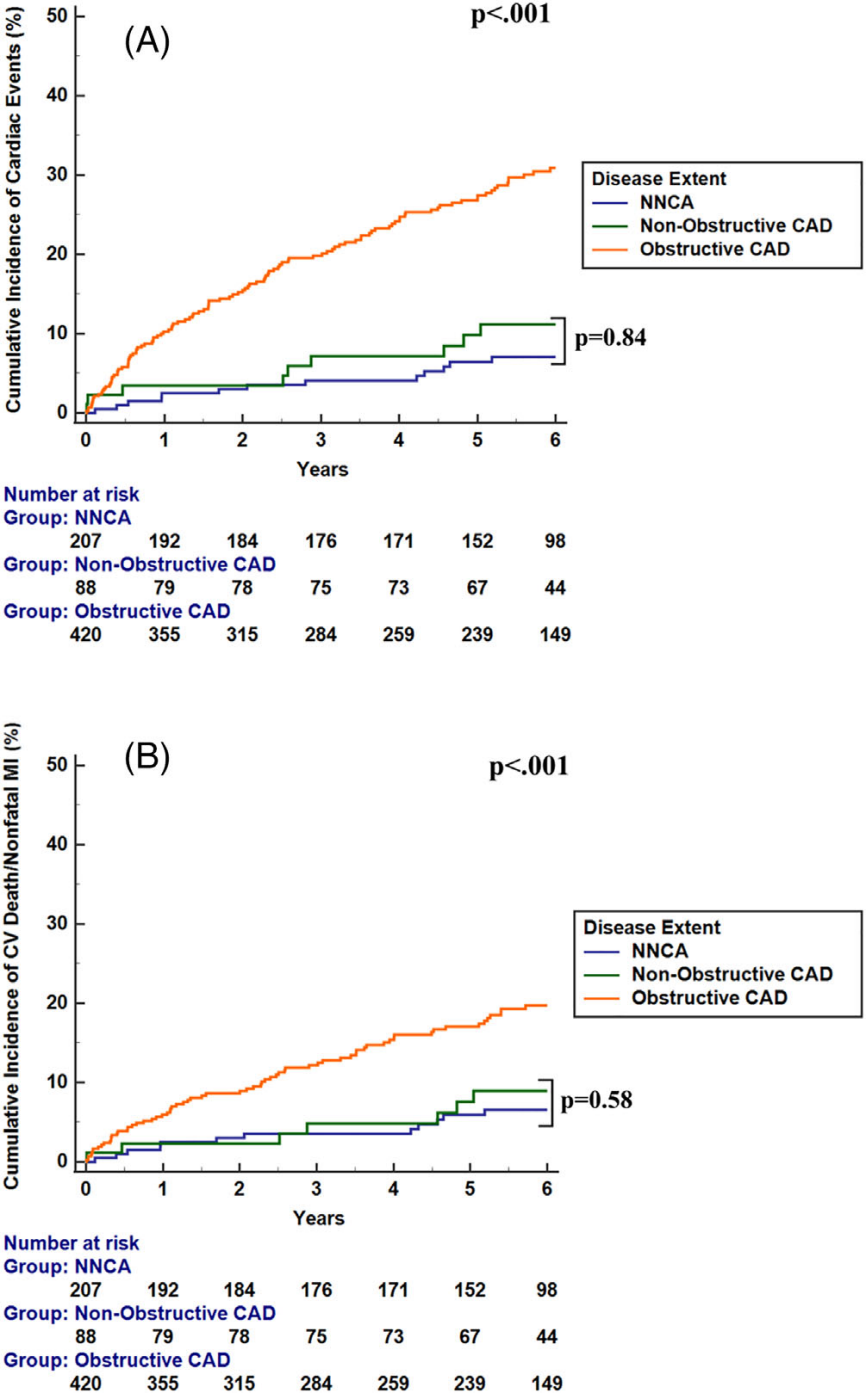
Cumulative events curves for cardiac death or nonfatal myocardial infarction (MI) stratified by stenosis grade at time of index catheterization in patients without HF (A). There was a higher risk of cardiac death for those with obstructive coronary artery disease (CAD) compared to those with normal or near‐normal coronary arteries (NNCA, *p* < .001) or nonobstructive CAD (*p* < .001). No difference in cardiac death or nonfatal MI was observed in patients with NNCA versus nonobstructive CAD (*p* = .84). Cumulative cardiac events curves stratified by stenosis grade at time of index catheterization in patients without HF (B). Cardiac events include cardiac death, nonfatal myocardial infarction, and late revascularization. There was a higher risk of cardiac events for those with obstructive CAD compared to those with normal or *p* < .001) or nonobstructive CAD (*p* < .001). No difference in cardiac events was observed in patients with NNCA versus nonobstructive CAD (*p* = .58). HF, heart failure

Event rates were then calculated in the 715 patients without HF (given the known attributable risk of HF to cardiovascular events). Results were similar to the event rates of the entire cohort. At a median of 6.0 years, there were significant decreases in survival free from CD and from cardiac events for those with obstructive CAD compared with patients with NNCAs or nonobstructive CAD (*p* < .001 for both). No differences between NNCA and nonobstructive CAD patients in rates of CD or NFMI (2.0 vs. 2.1%/year, *p* = .84) or cardiac events (2.4% vs. 2.9%/year, *p* = .58) were observed (Figure 3). Likewise, event rates were low, and again similar by gender (*p* = .70).

### 3.3 Medication usage at follow‐up

**FIGURE 4:**
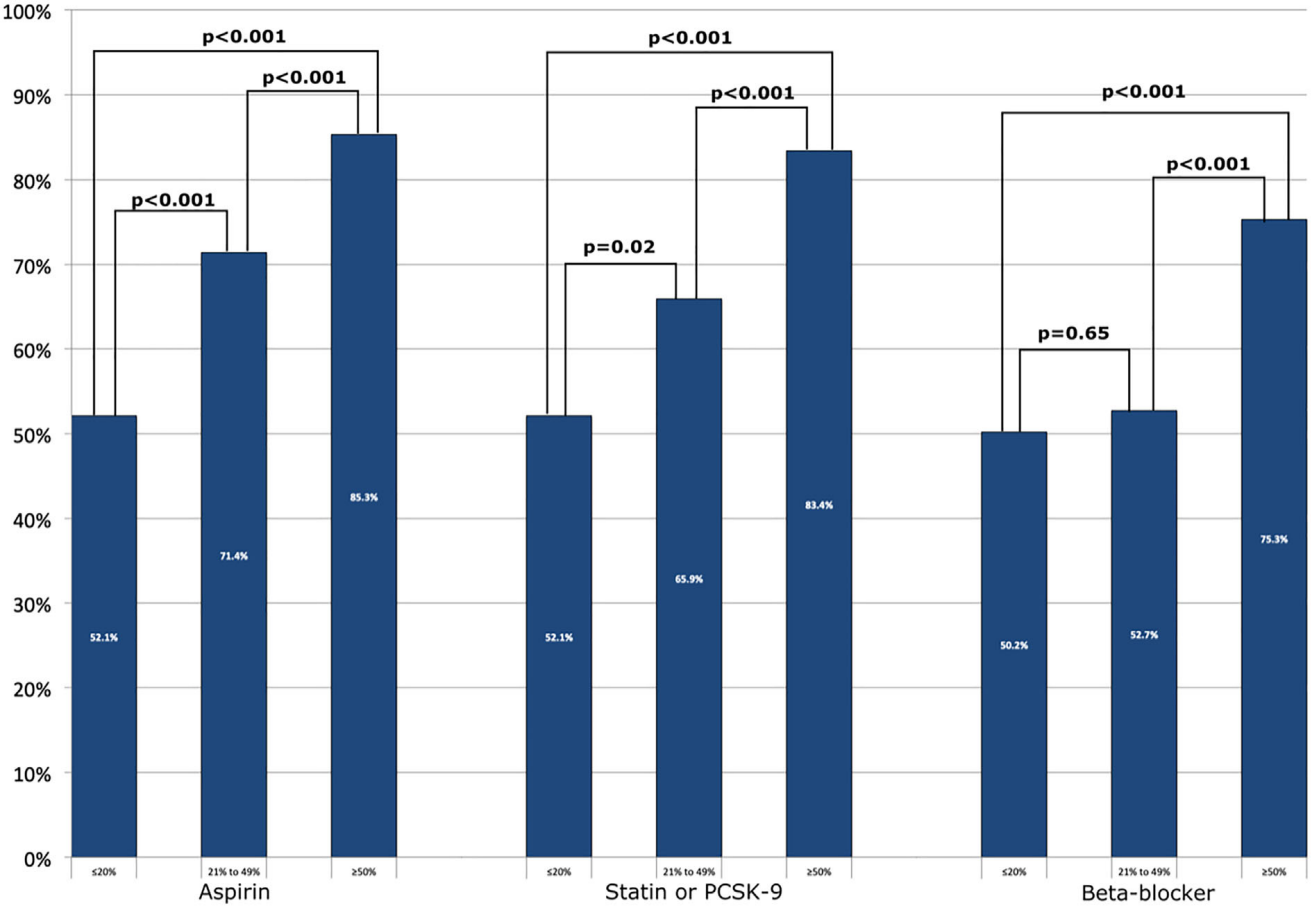
Percentage of patients on aspirin, lipid lowering therapy (statin or proprotein convertase subtilisin/kexin type 9 [PCSK‐9] inhibitors), and beta‐blocker therapy in patients without HF. The frequency of aspirin, lipid lowering therapy, and beta‐blocker therapy was increased in patients with nonobstructive coronary artery disease (CAD) compared to those with normal or near‐normal coronary arteries (NNCAs), *p* < .001 for all medications). Likewise, these medications were also more commonly prescribed in the obstructive CAD patients compared to those with nonobstructive CAD (*p* < .001 for all medications)

Medication data was available in all 850 patients with available follow‐up. The 715 patients referred without HF were analyzed separately due to differing goal‐directed medical therapy in nonischemic cardiomyopathy. Cardiac medication usage in patients without HF at time of ICA and at time of follow‐up is summarized in Table 2. At a median follow‐up of 6.0 years, there was an overall increase in guideline‐directed medical therapy for CAD with aspirin (54%–75%), lipid lowering therapy (48%–72%), and beta‐blocker therapy (40%–65%) (Figure 4). Treatment with these drugs were more commonly prescribed in the nonobstructive patients compared to the NNCAs (*p* < .001 for all medications) and likewise were also more commonly prescribed in the obstructive CAD patients compared to the nonobstructive CAD patients (*p* < .001 for all medications). In the subgroup with nonobstructive CAD, prevalence of aspirin therapy increased from 51% to 71%, and statin therapy from 52% to 66%. There were no significant differences in aspirin, statin or beta‐blocker usage by gender in patients with either NNCAs, nonobstructive CAD, or obstructive CAD (*p* > .05 for all) by chi‐square analysis.

**TABLE 2:** Study cohort medications subdivided by severity of angiographic coronary stenosis in patients without heart failure

Medication			Severity of coronary stenosis					
	Total cohort, n (%)		≤20% (n [%])		21%–49% (n [%])		≥50% (n [%])	
	Baseline	Follow‐up	Baseline	Follow‐up	Baseline	Follow‐up	Baseline	Follow‐up
Aspirin	397 (53.7)	551 (74.6)	90 (41.1)	114 (52.1)	46 (50.5)	65 (71.4)	257 (59.9)	366 (85.3)
Statin	357 (48.3)	532 (72.0)	86 (39.3)	114 (52.1)	47 (51.6)	60 (65.9)	218 (50.8)	358 (83.4)
PCSK‐9 inhibitors	0 (0)	7 (1.0)	0	0	0	0	0 (0)	7 (1.6)
Beta‐blocker	296 (40.1)	481 (65.1)	79 (36.1)	110 (50.2)	41 (45.1)	48 (52.7)	171 (39.9)	323 (75.3)

Abbreviation: PCSK‐9, proprotein convertase subtilisin/kexin type 9.

## 4 DISCUSSION

We previously reported the short‐term cardiac event rates (CD, NFMI infarction and late revascularization) in 925 consecutive patients who underwent non‐emergent ICA for either an abnormal stress test, stable or unstable angina, a NSTEMI or HF.^13^ Only 55.5% of these patients had obstructive coronary artery disease, with NNCA found in 31% and nonobstructive CAD found in 13.5%.^13^, ^24^ More women than men had NNCA or nonobstructive CAD. Pretest risk was high in 75% of the cohort. At a median follow‐up of 2 year, we found no difference in annual rates of CD or MI between those with NNCA (1.0%) and those with nonobstructive CAD (1.1%). No difference in outcomes between patients with NNCA and those with nonobstructive CAD was observed even in those with high‐risk ischemia on stress testing or a troponin >1.0 ng. The event rate was significantly higher in patients with obstructive CAD (6.7%). We found no sex differences in outcomes in any of the three groups. The finding that patients with nonobstructive CAD had a very low‐event rate, similar to that of patients with normal or NNCA, was unexpected, as was the low‐event rate in NSTEMI patients with nonobstructive CAD. In addition, women with nonobstructive CAD did not have a worse prognosis than men.

In the current study, we extended the follow‐up of this cohort of patients for a median of 6 years. Kaplan–Meier analysis again showed no difference between patients with normal or NNCA and nonobstructive CAD in rates of CD or NFMI or all cardiac events. In patients referred for ICA with either an abnormal stress test, stable or unstable angina or a NSTEMI, the annual rates of CD or NFMI were 2.0% in patients with NNCA versus 2.1% in those with nonobstructive CAD. As seen at short‐term follow‐up,^13^ the long‐term follow‐up reported in the present study still showed a higher subsequent event rate for patients with obstructive CAD. Men and women in all three anatomic groups still had similar event rates during the longer follow‐up period. Interestingly, patients with NSTEMI and nonobstructive CAD had the same risk of subsequent events as patients who underwent ICA for either an abnormal stress test, stable angina or unstable angina. The event rate was higher in patients who presented with HF and found to have nonobstructive CAD compared to patients undergoing angiography for the other indications.

Many prior studies have shown that symptomatic patients with nonobstructive CAD have a worse prognosis than patients with normal coronary arteries.^4^, ^5^, ^6^, ^7^ In a meta‐analysis of 54 studies from 1990 to 2015 comprising of patients with stable angina and NSTEMI, the risk of events was higher in patients with nonobstructive CAD (>20% and <50% stenosis) than in patients with normal or NNCA (≤20% stenosis).^4^ The death or NFMI rate was 0.3%/year in patients with normal or NNCA versus 0.7%/year in patients with nonobstructive CAD. For the NSTEMI patients with nonobstructive CAD in this meta‐analysis, the death and NFMI rates were 1.2%/year and 4.1%/year, respectively. In another meta‐analysis of patients with nonobstructive CAD comprising 64 905 patients the cardiac event rate was significantly higher in patients with nonobstructive CAD compared to patients with normal coronary arteries (3.17 odds ratio).^5^ A third meta‐analysis of studies employing CCTA demonstrated that the annual mortality was 0.74% in patients with nonobstructive CAD compared to 0.15% for patients with normal coronary arteries.^6^ In a large observational single center study of 11 000 patients undergoing ICA, those with nonobstructive CAD had a worse prognosis than patients with no CAD.^7^ The risk of cardiovascular events has been described to be related to the extent of nonobstructive CAD/plaque burden and the number of coronary vessels involved.^7^, ^8^, ^25^, ^26^ The more extensive the plaque burden in patients with nonobstructive CAD, the higher the subsequent cardiac event rate.^27^, ^28^, ^29^ Similarly, such patients exhibiting high‐risk plaque characteristics (e.g., low‐attenuation plaque, positive area remodeling) have an increased risk of cardiac events.^27^, ^30^, ^31^, ^32^

In the SCOT‐HEART study, approximately one‐half of subsequent MIs occurred among patients who had nonobstructive CAD at baseline.^33^ In the PROMISE trial, two‐thirds of subsequent cardiac events occurred in patients with nonobstructive CAD.^3^ CCTA registry data are consistent with these observations.^34^, ^35^ Thus, these studies clearly indicate that for patients with nonobstructive CAD, although associated with a lower incidence of future cardiac events compared to patients with obstructive CAD, the risk for a future ACS or progression of an ischemic chest pain syndrome is significant. Although stress SPECT MPI or stress echocardiography may not identify such patients with nonobstructive CAD, abnormal coronary flow reserve measurements may be revealed by quantitative PET or CMR.^11^

In contrast to the findings of these prior studies cited above showing a higher risk for cardiac events for patients with nonobstructive CAD compared to patients with angiographic normal or NNCA, we found no difference in NFMI or CD, NFMI or late revascularization in patients with nonobstructive CAD versus those with NNCA. Additionally, this excellent long‐term prognosis extended to the subgroup of patients with nonobstructive CAD who underwent ICA following a NSTEMI. The outcomes for this group were similar to outcomes in patients who underwent invasive evaluation for CAD after an abnormal stress test, stable angina or unstable angina. The latter finding in NSTEMI patients with a troponin elevation prior to coronary angiography was unexpected since it was the common belief that such patients have a worse prognosis than chest pain patients without a troponin elevation who are referred for ICA. We speculate that statin and aspirin use in this population may have led to improved outcomes. However, this finding could be due to the mislabeling of some patients classified as having truly “normal” coronary arteries. An inherent limitation of conventional ICA is the inability to assess extraluminal plaque. CCTA provides better evaluation of extraluminal plaque and many of our patients with “normal or near normal” coronary arteries likely had some nonobstructive CAD if imaged by CCTA prior to ICA; therefore, some of the patients with normal or near normal designation by ICA could have been labeled as having varying degrees of nonobstructive CAD by CCTA. Of note, the recently published VERDICT trial demonstrated CCTA to have a high‐diagnostic accuracy to rule out obstructive CAD (>50% stenosis) in patients presenting with NSTEMI.^36^ CCTA may play a future role in risk stratifying NSTEMI patients prior to ICA to identify those with obstructive CAD and to diagnose nonobstructive atherosclerosis, which can then be further optimized by medical therapy. Furthermore, coronary vasospasm could have played a role in NSTEMI is some patients with nonobstructive CAD, which was not evaluated in this cohort. Recent advances in optical coherence tomography have demonstrated the ability to evaluate the presence and characteristics in plaques and other coronary pathologies to allow for risk stratification in this population.^37^

With respect to gender, we found no difference in either mortality, the rate of CD or NFMI or all cardiac events between men and women with nonobstructive CAD or obstructive CAD. Some prior studies suggested that women with nonobstructive CAD may have a worse prognosis than men.^11^, ^12^

One possible explanation for the excellent prognosis in patients with nonobstructive CAD in our study may relate to enhancement of medication usage for secondary prevention after the invasive diagnostic study. Excluding patients initially referred for ICA for HF, 75% of the remaining group was taking aspirin and 73% were taking a statin or a PCSK‐9 inhibitor at the time of follow‐up. With respect to the subgroup with nonobstructive CAD in our study, 71% were taking aspirin and 66% were taking a statin at follow‐up. This compared to 85% and 83% of patients with obstructive CAD who was taking aspirin and a statin, respectively, at follow‐up. There were no gender differences in medication usage.

Other investigators have proposed that patient and physician knowledge of coronary anatomy after either ICA or CCTA may guide more appropriate guideline‐based therapy for patients shown to have coronary atherosclerosis, even though not obstructive. Pooled data from the PROMISE and SCOT‐HEART registries comprising 86 000 patients revealed a 30% reduction in risk of events when management was guided by CCTA versus traditional care.^38^ In another study, annual all‐cause mortality was 0.46% in statin users versus 1.40% in nonusers in patients found to have nonobstuctive CAD on CCTA.^26^ A post‐hoc analysis of the SCOT‐HEART trial, statin use increased from 43% to 50% in the standard care group at 1 year, whereas it rose from 44% to 59% in the CCTA group.^38^ The investigators of the SCOT‐HEART found that the major difference in statin usage between the two arms of the study was the identification of nonobstructive CAD in the CCTA group, for which enhanced therapy was prescribed.^33^ The association between statin therapy and better clinical outcomes in more than 8000 patients with nonobstructive CAD was observed regardless of age, sex, presence of hypertension or diabetes, coronary artery calcium score, low‐density lipoprotein cholesterol levels, or renal function.^39^

The findings in the present study regarding changes in medication usage after identification of nonobstructive CAD are consistent with the findings of studies cited above. The major difference between our study using ICA to detect CAD, rather than CCTA, was a greater post‐procedure increase in aspirin and statin usage. Nearly 70% of patients with nonobstructive CAD were taking statins and aspirin at a median of 6 years of follow‐up. For patients with obstructive CAD, approximately 85% of patients were on these medications.

No gender differences in prescribing aspirin or statins were observed. This is in contrast to the findings of another recently reported study in patients with nonobstructive CAD identified after ICA, in which women were less likely than men to report statin use at follow‐up.^7^

The cardiac event rates observed for patients with NSTEMI who were found to have either normal or NNCA or nonobstructive CAD at angiography. Interestingly, the event free survival was similar to patients referred to ICA after an abnormal stress test, stable angina, or unstable angina. The annual death or nonfatal infarction rate in this subgroup of NSTEMI patients was only 2.6%/year and 2.0%/year, respectively. It is possible, although speculative, that this low‐event rate may have been due to the benefits of guideline‐directed medical therapy after NSTEMI.

### 4.1 Limitations of the study

There are several limitations of this study that deserve mention. First, it is a single‐center study with a limited number of patients in the nonobstructive CAD group, which may limit the ability to detect small statistical differences with low‐event rates. Furthermore, the limited number of patients may also limit our ability to detect statistical differences between outcomes stratified by referral indication (NSTEMI, abnormal stress testing, etc.) between patients with NNCAs and nonobstructive CAD. The study was retrospective in design. FFR was not performed routinely in all patients. Severity of CAD was solely assessed by quantitative coronary angiography. Approximately 8% of the original study cohort was lost to follow‐up, although the clinical characteristics of those lost to follow‐up were similar to the group for which follow‐up data was obtained. Another limitation of this study is the lack of reported cholesterol levels at the time of follow‐up.

## 5 CONCLUSIONS

We present long‐term follow‐up data in a cohort of consecutive patients without known prior CAD who underwent non‐emergent ICA for detection of CAD. A major finding of the current study is that patients who were found to have nonobstructive CAD, defined as 21%–49% stenosis, had similar low‐event rates to patients found to have normal or NNCA at angiography at a median of 6 years of follow‐up. Patients with NSTEMI and either normal or NNCA or nonobstructive CAD had a prognosis similar to patients referred for coronary angiography for chest pain or following an abnormal stress test. No gender differences were observed in outcomes regardless of angiographic classification. We speculate that these low‐event rates may be secondary to enhanced medical therapy after the detection of coronary plaque at angiography, albeit nonobstructive. This is consistent with what has been observed for patients with nonobstructive CAD in studies using CCTA.

## CONFLICT OF INTEREST

Dr. Bourque is a consultant for Pfizer and GE Healthcare. The other authors have nothing to disclose.

## AUTHOR CONTRIBUTIONS

Christopher A. Hanson: conceptualization, methodology, formal analysis, investigation, writing–original draft. Edwin Lu: formal analysis, investigation, visualization, writing–review and editing. Saad S. Ghumman: conceptualization, methodology, investigation. Michelle L. Ouellette: conceptualization, methodology, investigation. Adrián I. Löffler: conceptualization, methodology, investigation. George A. Beller: conceptualization, methodology, supervision, writing–review and editing. Jamieson M. Bourque: conceptualization, methodology, formal analysis, investigation, supervision, writing–review and editing.

## Supporting information

**Supplementary Table I**: Study cohort baseline characteristics in total and subdivided by severity of angiographic coronary stenosis in patients without heart failure

**Supplementary Table II**: Annual event rates for cardiac death, cardiovascular events, and cardiac death + nonfatal myocardial infarction (MI)

## Data Availability

The data that support the findings of this study are available on request from the corresponding author. The data are not publicly available due to privacy or ethical restrictions.
