# Supplementary material for: Long‐term outcomes in patients with normal coronary arteries, nonobstructive, or obstructive coronary artery disease on invasive coronary angiography
Source: Clin Cardiol. 2021 Jul 3;44(9):1286–95. doi: 10.1002/clc.23686 (PMC8428062; doi:10.1002/clc.23686)
Supplement: Supplementary file 1 — Supplementary Table I: Study cohort baseline characteristics in total and subdivided by severity of angiographic coronary stenosis in patients without heart failure [file CLC-44-1286-s001.docx]

**Supplementary Table I:** Study Cohort Baseline Characteristics in Total and Subdivided by Severity of Angiographic Coronary Stenosis in Patients without Heart Failure.

|  | | | | |
| --- | --- | --- | --- | --- |
| Clinical Characteristic | Total Cohort, n (%) | Severity of Coronary Stenosis | | |
|  |  | ≤20%  (n (%)) | 21% to 49%  (n (%)) | ≥50%  (n (%)) |
| Total Patients | 715 | 207 (29.0) | 88 (12.3) | 420 (58.7) |
| Age, years* | 62.2 (±11.5) | 57.5 (±12.0) | 63.9 (±9.8) | 64.1 (±11.6) |
| Female | 332 (46.4) | 118 (57.0) | 52 (59.1) | 162 (38.6) |
| Caucasian | 608 (85.0) | 165 (81.2) | 75 (85.2) | 368 (87.6) |
| Diabetes mellitus | 248 (34.7) | 62 (30.0) | 28 (31.8) | 158 (37.6) |
| Hypertension | 584 (81.7) | 150 (72.5) | 69 (78.4) | 365 (86.9) |
| Hyperlipidemia | 512 (71.6) | 126 (60.9) | 52 (59.0) | 334 (7.5) |
| Tobacco use | 233 (32.6) | 62 (30.0) | 24 (27.3) | 147 (35.0) |
| BMI ≥30 | 345 (48.3) | 118 (57.0) | 38 (43.2) | 189 (45.0) |
| Peripheral vascular disease | 93 (13.0) | 14 (6.8) | 8 (9.1) | 71 (16.9) |
| Cerebrovascular disease | 25 (3.5) | 2 (1.0) | 2 (2.3) | 21 (5.0) |
| Chronic kidney disease grade ≥3 | 66 (9.2) | 12 (5.8) | 6 (6.8) | 48 (11.4) |
| ASCVD risk ≥7.5% | 480 (67.1) | 112 (54.1) | 60 (68.2) | 308 (73.3) |
| Chest pain or anginal SOB | 635 (88.8) | 182 (87.9) | 69 (78.4) | 384 (91.4) |

Table 1 legend: Baseline characteristics of the study cohort. BMI = body mass index. ASCVD risk was unable to be classified in 120 patients. * Mean ± standard deviation provided for continuous variables.
