# Supplementary material for: Long‐term outcomes in patients with normal coronary arteries, nonobstructive, or obstructive coronary artery disease on invasive coronary angiography
Source: Clin Cardiol. 2021 Jul 3;44(9):1286–95. doi: 10.1002/clc.23686 (PMC8428062; doi:10.1002/clc.23686)
Supplement: Supplementary file 2 — Supplementary Table II: Annual event rates for cardiac death, cardiovascular events, and cardiac death + nonfatal myocardial infarction (MI) [file CLC-44-1286-s002.docx]

**Supplementary Table II:** Annual Event Rates for Cardiac Death, Cardiovascular Events, and Cardiac Death + Non-Fatal Myocardial Infarction (MI).

|  | | | | | | |
| --- | --- | --- | --- | --- | --- | --- |
|  | Male  Severity of Coronary Stenosis | | | Female  Severity of Coronary Stenosis | | |
| Events | ≤20% | 21% to 49% | ≥50% | ≤20% | 21% to 49% | ≥50% |
| Cardiac Death | 1.36% | 1.25% | 1.77% | 1.16% | 0.53% | 2.44% |
| Cardiovascular Events | 1.79% | 2.58% | 5.22% | 1.68% | 1.95% | 7.00% |
| Cardiac Death + Non-Fatal MI | 1.49% | 2.18% | 2.99% | 1.62% | 1.60% | 3.51% |

Supplementary Table I legend: Annual event rate for cardiac death, cardiovascular events, and combined cardiac death and non-fatal MI divided by gender and further subdivided by severity of angiographic coronary stenosis.
